# Extended resection in pancreatic metastases: feasibility, frequency, and long-term outcome: a retrospective analysis

**DOI:** 10.1186/s12893-015-0114-1

**Published:** 2015-12-11

**Authors:** Georg Wiltberger, Julian Nikolaus Bucher, Felix Krenzien, Christian Benzing, Georgi Atanasov, Moritz Schmelzle, Hans-Michael Hau, Michael Bartels

**Affiliations:** Department of Visceral, Transplantation, Thoracic, and Vascular Surgery, University Hospital Leipzig, 04103 Leipzig, Germany; Department of Surgery, University Hospital Großhadern (LMU), Munich, Germany; Department of General, Visceral, and Transplant Surgery, Charité - Universitätsmedizin Berlin, Campus Virchow Klinikum, Augustenburger Platz 1, 13353 Berlin, Germany

**Keywords:** Multivisceral resection, Metastases to the pancreas, Pancreaticoduodenectomy

## Abstract

**Background:**

Metastases to the pancreas are rare, accounting for less then 2 % of all pancreatic malignancies. However, both the benefit of extended tumor resection and the ideal oncological approach have not been established for such cases; therefore, we evaluated patients with metastasis to the pancreas who underwent pancreatic resection.

**Methods:**

Between 1994 and 2012, 676 patients underwent pancreatic surgery in our institution. We retrospectively reviewed patients’ medical records according to survival, and surgical and non-surgical complications. Student’s *t*-test and the log-rank test were used for statistical analysis.

**Results:**

Eighteen patients (2.7 %) received resection for pancreatic metastases (12 multivisceral resections and 6 standard resections). The pancreatic metastases originated from renal cell carcinoma (*n* = 10), malignant melanoma (*n* = 2), neuroendocrine tumor of the ileum (*n* = 1), sarcoma (*n* = 1), colon cancer (*n* = 1), gallbladder cancer (*n* = 1), gastrointestinal stromal tumor (*n* = 1), and non-small cell lung cancer (*n* = 1). The median time between primary malignancy resection to metastasectomy was 83 months (range, 0–228 months). Minor surgical complications (Grade I-IIIa) occurred in six patients (33.3 %) whereas major surgical complications (Grade IIIb-V) occurred in three patients (16.6 %). No patients died during hospitalization. The median follow-up was 76 months (range, 10–165 months). One-year, 3-year and 5-year survival for standard resection versus multivisceral resection was 83, 50, and 56 % versus 83, 66, and 50, respectively. Twelve patients died after a median of 26 months (range, 5–55 months).

**Conclusions:**

A surgical approach with curative intent is justified in select patients suffering from metastases to the pancreas and offers good long-term survival. The resection of pancreatic metastases of different tumor types was associated with favorable morbidity and mortality when compared with resection of the primary pancreatic malignancies. Our findings also demonstrated that multivisceral resection was feasible, with acceptable long term outcomes, even though morbidity rates tended to be higher after multivisceral resection than after standard resection.

## Background

Metastases to the pancreas are rare and account only for 1–2 % of all pancreatic malignancies [1]. Most primaries that spread to the pancreas are renal cell carcinomas (RCC), lung cancers, malignant melanomas, and malignancies of the gastrointestinal tract [2, 3]. However, at the time of diagnosis, patients often present with widespread systemic disease and therefore, no curative treatment is applicable. Several studies have demonstrated survival benefit and improved quality of life after complete metastasectomy for isolated lung or liver metastases [4, 5]. Therefore, extended surgical intervention is a well-established approach in a multidisciplinary concept for select patients suffering from colorectal or pulmonary metastases in the liver [6]. However, extended surgery for pancreatic metastasectomy is rare and remains debatable as there have been few studies on the procedure, all of which have reported controversial results [7–9]. Patients with localized extrapancreatic disease appear to be suitable for pancreatic resection but the ideal oncological approach has not been established and the benefit of multivisceral resection (MVR) remains undetermined. Our aim was to assess the frequency and feasibility of MVR for metastases to the pancreas. We also analyzed the influence of MVR on perioperative and long-term outcomes compared with standard resection.

## Methods

We retrospectively analyzed the medical records of patients who underwent pancreatic resection at the Department of Visceral, Transplant, Thoracic, and Vascular Surgery, University Hospital Leipzig, Leipzig, Germany, between 1994 and 2012.

For this study ethical approval was obtained from the institutional local ethical committee (AZ 318-14-06102014, Ethical committee of the University Clinic Leipzig, Leipzig). Due to the retrospective design of the study and accordingly national guidelines, the local ethic committee confirmed, that informed consent was not necessary from participants.

All patients were operated on with curative intent. We included patients with extrapancreatic spread in this study only when extrapancreatic disease appeared to be resectable, and we excluded patients with primary tumors that had infiltrated the pancreas through direct extension.

We assessed the following patient characteristics: sex, age, body mass index, preoperative symptoms, comorbidities, type of resection, duration of operation, required units of fresh frozen plasma and/or packed red blood cells, median length of stay on the intensive care unit, total length of stay, time between surgery for the primary tumor and pancreatic resection, postoperative morbidity according to Clavien-Dindo classification [10], presence of pancreatic fistulae according to the International Study Group for Pancreatic Fistula criteria [11], hospital mortality defined as death within the first 60 days after resection, overall survival rate, disease-free survival, and histopathological data. Patients who died within the first 90 days after resection were excluded from further statistical analysis. MVR was defined as resection of one additional organ, excluding the spleen.

### Statistics

Data are presented as median (range) unless otherwise specified. Statistical differences between groups were determined by Student’s *t*-test. Student’s *t*-test and the log-rank test were used to analyze continuous variables and overall survival excluding in-hospital mortality, respectively. All statistical analyses were performed with SPSS for Windows (version 12.0, SPSS Inc., Chicago, IL, USA).

## Results

### Patient characteristics and preoperative symptoms

Between 1994 and 2012, 676 patients were scheduled for pancreatic surgery, of which 18 (2.6 %) received resection for metastatic disease of the pancreas (Table 1). Eight of the patients were men and 10 were woman with a median age of 65 years (range, 22–75 years) and a median body mass index of 25.4 kg/m^2^ (range, 18–31 kg/m^2^). Four patients (22.2 %), had diabetes mellitus type II and 13 (72.2 %) patients received medication for high blood pressure (Table 2). At the time of diagnosis of pancreatic metastases, eight patients (44.4 %) had one or more tumor-related symptoms including weight loss (*n* = 8), abdominal pain (*n* = 8), decrease in general performance (*n* = 5), obstructive jaundice (*n* = 2), or absence of appetite (*n* = 1). Ten patients (55.5 %) had no specific symptoms and the diagnosis of pancreatic metastases was obtained through routine follow-up examinations.

**Table 1:** Patient characteristics

Case No.	Primary Malignancy	Location in the pancreas (sync./metac.)	Time interval (months)*	Type of Operation	further metastases at detection of pancreatic metastasis□	tumor recurrence/survival status^†^
1	RCC	Head (sync.)	0	PPPD + Nephrectomy	no	no/alive
2	Lung-Cancer	Cauda (metac.)	83	DP + Gastrectomy + Splenectomy + vertebral body resection	Vertebral body (s)	no/TRD
3	RCC	Head (metac.)	142	PPPD	no	no/Non-TRD
4	RCC	Head (sync.)	0.8	PPPD	no	Local recurrence/alive
5	RCC	Head/Corpus/Cauda (metac.)	120	Enucleation in pancreatic head + DP + Splenectomy	Lung (s)	Thyroid/TRD
6	RCC	Head/Corpus/Cauda (metac.)	132	TP + Spleenectomy + distal Gastrectomy	Thyroid (s)	no/TRD
7	Gallblader-Cancer	Head (sync.)	0	PPPD + Liver resection (SII SIII)	Liver (a)	yes/TRD
8	RCC	Corpus/Cauda (sync.)	17	DP + Spleen + Colon +Jejunom	Pulmo (s)	no/TRD
9	GIST	Head (metac.)	34	PPPD + Hemicolectomy	Liver (a)	yes/alive
10	RCC	Head (metac.)	106	Whipple	no	Lung/TRD
11	Sarcoma	Head (sync.)	0	Whipple + Hemihepatectomy + Hemicolectomy	Liver (s)	Liver/TRD
12	RCC	Head/Corpus/Cauda (metac.)	66	TP + Spleen	no	Cerebral/TRD
13	Melanoma	Head/Corpus/Cauda (metac.)	90	TP + Segemental liver resection	Liver (a)	Liver/alive
14	RCC	Cauda (metac.)	123	DP	Pulmo (s)	Thyroid/TRD
15	Melanoma	Head (metac.)	228	PPPD + Hemihepatectomy	Liver (a)	Liver/TRD
16	RCC	Head (sync.)	0	DP + Spleen + Nephrctomy	no	no/alive
17	Colon-Cancer	Head (metac.)	29	PPPD + Liver resection (Lobus caudatus)	Liver (s)	no/alive
18	NET Ileum	Head (sync.)	78	PPPD + Jejunum	Jejunum (s)	no/alive

sync. indicates synchronous metastases to the pancreas; metac. indicates metachronous metastases to the pancreas; * interval from resection of primary tumor to resection of pancreatic metastasis; □ excluding pancreas (a, after; s, synchronous; p, prior); † at time of study; RCC indicates renal cell carcinoma; NET indicates neuroendocrine tumor; PPPD indicates pylorus-preserving pancreaticoduodenectomy; DP indicates distal pancreatectomy; TP indicates total pancreatectomy; TRD indicates tumor-related death

**Table 2:** Demographic data

Characteristics	Total (*n* = 18)	Standard Resection (*n* = 6)	Multivisceral resection (MVR; *n* = 12)	*p*-value*
Age (median; Range)	65 (22–75)	70 (49–75)	58 (22–72)	0.074
Gender (%)				
Female	10 (55.6)	4 (22.2)	6 (33.3)	0.548
Male	8 (44.4)	2 (11.1)	6 (33.3)	0.548
BMI (median; Range)	25.4 (18–31.1)	26 (21.7–30.4)	24.4 (18–31.1)	0.264
Comorbidities (%)				
Diabetes mellitus	4 (22.2)	2 (11.1)	2 (11.1)	0.932
Arterial hypertension	13 (72.2)	5 (33.3)	8 (44.4)	0.817
Metabolic syndrome	3 (16.7)	1 (5.6)	2 (11.1)	0.932
COPD*	2 (11.1)	0 (0)	2 (11.1)	0.104
CAD	5 (27.8)	1 (5.6)	4 (22.2)	0.374
Preoperative symptoms (%)				
* Asymptomatic*	10 (55.6)	4 (22.2)	6 (33.3)	0.548
* Symptomatic*				
Weight loss	8 (44.4)	3 (16.7)	5 (33.3)	0.984
Abdominal pain	7 (38.9)	3 (16.7)	4 (33.3)	0.682
obstructive jaundice	2 (11.1)	0 (0)	2 (11.1)	0.103
decrease in general performance	3 (16.7)	1 (5.6)	2 (11.1)	0.742
absence of appetite	1 (5.6)	1 (5.6)	0 (0)	0.170
Sleep hyperhidrosis	1 (5.6)	0 (0)	1 (5.6)	0.166
New onset of diabetes	1 (5.6)	0 (0)	1 (5.6)	0.166

COPD indicates chronic obstructive pulmonary disease; CAD indicates coronary artery disease

### Primary tumor characteristics

The majority of pancreatic metastases originated from renal cell carcinoma (*n* = 10; 55.5 %), malignant melanoma (*n* = 2), and neuroendocrine tumor of the ileum (*n* = 1). Other primaries included sarcoma (*n* = 1), colon cancer (*n* = 1), gallbladder cancer (*n* = 1), gastrointestinal stromal tumor (*n* = 1), and non-small cell lung cancer (*n* = 1). Metastases to the pancreas were located mainly in the pancreatic head (*n* = 10) followed by the total pancreas (*n* = 3) and the cauda region (*n* = 3). In two patients, metastases were simultaneously located in the corpus and cauda region. Eleven patients (61 %) had simultaneous metastases in other organs and seven of these patients underwent simultaneous resection of the extrapancreatic masses. The other four patients received a subsequent procedure (metastasectomy of the lungs in three cases and one thyroidectomy). In four patients, the diagnosis of pancreatic metastases coincided with that of the primary malignancy, which was RCC in all cases. In three of these patients, primary tumor and metastatic resection was performed as one procedure. In one patient, an intraoperative biopsy of the pancreatic head during tumor-nephrectomy confirmed metastatic disease and metastasectomy was performed four weeks later. In three patients (30 %) with RCC multifocal lesions in the resected specimens were detected.

### Surgical procedures

The median time between resection of the primary malignancies to resection of the pancreatic metastases was 83 months (range, 0–228 months). The most frequently used surgical procedures were Whipple-procedure/pylorus-preserving pancreaticoduodenectomy (PPPD) in 10 patients and distal pancreatectomy (DP) in five patients. In four patients, DP was performed with and, in one patient, without splenectomy. In three patients, total pancreatectomy was performed, with simultaneous splenectomy in two cases. MVR was performed in 12 (66.6 %) patients. Five patients who received Whipple-procedure/PPPD or total pancreatectomy also required additional liver resection to remove synchronous extrapancreatic metastases. Two of these patients underwent additional bowel resection and three patients received additional gastric or bowel resection. In two patients who underwent PPPD or DP, simultaneous nephrectomy was performed and one patient received simultaneous vertebral body resection for solitary bone metastasis. The median operation time for all procedures was 322 min (range, 193–591 min). Ten patients received intraoperative fresh frozen plasma (median 3, range 0–18 units) and/or packed red blood cells (median 2, range, 0–10 units). Microscopically-free resection margins (R0) were achieved in 77.7 % of the patients.

### Perioperative outcome

Length of stay in the intensive care unit and total length of stay were 2 days (range, 1–50 days) and 21.5 days (range, 12–55 days), respectively (Table 3). No patients died during hospitalization. Postoperative complications occurred in 11 patients (61.1 %), of which 81 % were surgical and 18 % were non-surgical complications. Minor surgical complications (Grade I-IIIa, *n* = 6) were: new onset of diabetes in two patients; wound infection (*n* = 2), which was managed conservatively; and pancreatic fistula grade B with prolonged use of abdominal drainage (*n* = 2). Major surgical complications (Grade IIIb-V, *n* = 3) were: leakage from the hepaticojejunostomy site with the need for relaparotomy, bowel perforation with the need for relaparotomy, biliary leakage after combined liver and pancreas resection that had to be treated with an interventional drain, and a wound infection that required re-operation under general anesthesia, with each complication occurring in one case. Major non-surgical complications (Grade IIIb-V, *n* = 2) occurred in two patients and both developed pneumonia requiring readmission to the intensive care unit.

**Table 3:** Perioperative data

Data	Total (*n* = 18)	Standard Resection (*n* = 6)	Multivisceral resection (*n* = 12)	*p*-Value
Operative Data				
Length of operation (min)	322 (193–591)	261 (193–462)	346 (216–591)	0.137
FFPs and/or pRBCs	3 (0–18) 2 (0–10)	3 (0– 3) 1 (0–8)	3 (0–18) 4 (1–10)	0.291/0.838
Perioperative Data				
LOS-ICU	2 (1–50)	1 (1–3)	3 (1–50)	0.205
T-LOS	21 (12–55)	20 (16– 2)	23 (12–55)	0.898
Time interval*	72 (0–228)	113 (0–142)	31.5 (0–228)	0.259
Follow-up	76 (10–165)	59 (28–95)	53 (10–165)	0.104
1-year/3-year/5-year survival	84/66/55	83/50/56	83/66/50	
Histopathological data				
RO/R1/R2	15/3/0 (83.3/16.7/0)	7/0/0 (38.9/0/0)	8/3/0 (44.4/16.7/0)	0.081
Negative/positive LN	13/5 (72.2/27.8)	6/1 (33.3/5.6)	7/4 (38.9/22.2)	0.278
Postoperative Complications (Clavien-Dindo classification)				
*Non-Surgical related complications*				
Minor (Grade I-IIIa)	0 (0)	0 (0)	0 (0)	0
Major (Grade IIIb – IV)	2 (11.1)	1 (5.6)	1 (5.6)	0.681
*Surgical related complications*				
Minor (Grade I-IIIa)	6 (33.3)	2 (11.1)	4 (22.2)	0.781
Major (Grade IIIb – IV)	3 (16.7)	1 (5.6)	2 (11.1)	0.932
Overall Morbidity				
Mortality (60 days)	0 (0)	0 (0)	0 (0)	0
Overall-Mortality				
Follow up				
Local recurrence	1 (5.6)	1 (5.6)	0 (0)	0.166
Extrapancreatic Recurrence	9 (50)	4 (22.2	5 (33.3)	0.565
Further surgical interventions	6 (33.3)	2 (11.1)	4 (22.2)	0.781

FFP, fresh frozen plasma; pRBC, packed red blood cells; LOS-ICU, length of stay in intensive care unit; T-LOS; total length of stay

### Follow-up and survival

No patient was lost to follow-up and the median follow-up time was 76 months (range, 10–165 months) with 12 patients dying after a median of 26 months (range, 5–55 months). Death was tumor-related in 11 cases and non-tumor-related in one case. During follow-up, nine patients suffered from extrapancreatic recurrence. Five patients received further surgical treatment including thyroidectomy, liver resection, or metastasectomy of the lungs. In one patient, cerebral metastasis was treated by stereotactic irradiation. Local recurrence of pancreatic disease was seen in one patient and was successfully controlled by total resection of the pancreatic remnant.

**Fig. 1:**
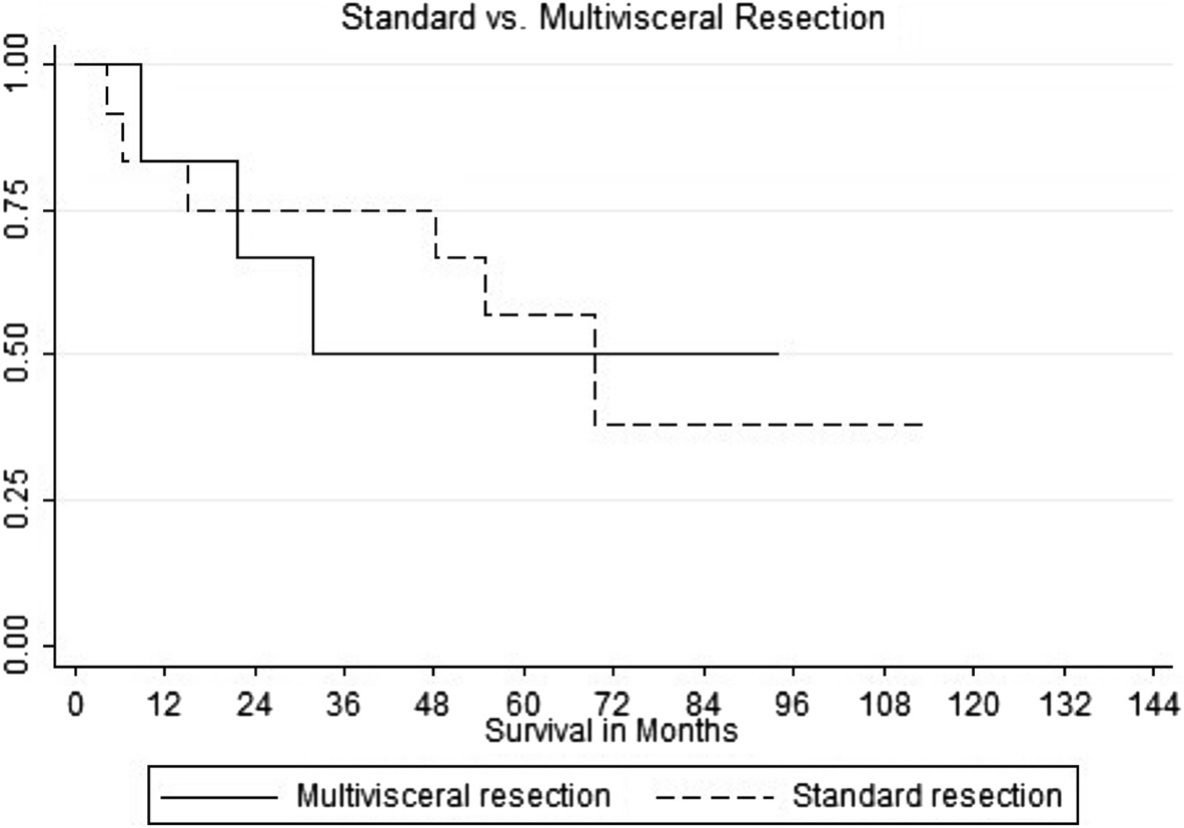
Kaplan–Meier survival curve showing survival for patients who underwent standard resection (solid line) versus multivisceral resection (dotted line) (in months)

In our sample, the time interval between resection of the primary malignancy and detection of metastatic disease did not correlate significantly with overall survival after resection of the pancreatic metastases. Kaplan-Meier curve for survival with standard resection versus MVR are shown in Fig. 1. One-, 3- and 5-year survival for standard resection versus MVR was 83, 50, and 56 %, versus 83, 66, and 50 %, respectively.

## Discussion

Surgical interventions for metastatic disease have increased over the last decade, concurrent with considerable improvement in quality of life and long-term survival of up to 10 years following resection for the most common (liver or lung) metastases [12, 13]. As a result, surgical resection of metastases is now an integral part of a multidisciplinary oncological approach [6]. In contrast, metastases to the pancreas are uncommon and the majority of patients present with no specific symptoms [14] and with non-resectable widespread disease at the time of diagnosis. However, in cases of isolated disease, surgical intervention may be beneficial in terms of overall survival, even in patients with localized extrapancreatic metastases, and in terms of the need for MVR to obtain complete tumor-free resection margins.

Based on our experience, surgical intervention is justified in select patients diagnosed with metastases to the pancreas. Our data showed that surgical resection for pancreatic metastases is feasible and provides good long term results, even in patients undergoing MVR. Perioperative morbidity and in-hospital mortality were comparable to studies evaluating standard pancreatic resection for primary malignancies [15]. In our study, morbidity after MVR tended to be higher than after standard resection. However, MVR for pancreatic metastases should not be considered an absolute contraindication for surgery, because our results indicated equivalent overall survival in the MVR group.

The limitations of our study are the small cohort and the absence of a control group treated by other therapeutic strategies. Therefore, general recommendations cannot be made based on our data, even though complete resection (R0) is generally considered a good prognostic marker for patients’ overall survival [16]. For instance, long-term survival of 10 years can be seen after complete resection of colorectal liver metastases, which emphasizes the importance of this therapeutic option [12]. In our study, the rate of microscopically-free resection margins (77.7 %) did not differ from other studies reporting positive resection margins for pancreatic cancer of 17–30 % [17–19].

Because of the high incidence of metastatic disease originating from RCC, the most valid conclusions can be provided for this tumor type. Consequently, most reported data refer to RCC and are comparable with our results in terms of incidence and overall survival [1, 8, 20]. In our study, 10 patients (55.5 %) had pancreatic metastases from RCC and the overall survival of this subgroup was 60 %. In a recently published review, Tanis et al. reported a 5-year survival rate of 72.6 % in 311 cases following pancreatic surgery for RCC metastases [21]. In this context, time of metastatic onset is discussed as a prognostic marker for long-term survival. A small number of studies have observed a trend to better overall survival in patients with long disease-free interval when evaluating primary tumor resection and onset of metastases to the pancreas. We did not see a similar effect in our patients because overall survival did not differ significantly between patients with longer disease-free interval. These findings are supported by the results of a meta-analysis which identified 15 studies addressing pancreatic metastasectomy for RCC [1]. In the univariate analysis, time from resection of the primary tumor did not affect overall survival.

Many changes have been made, regarding the oncological treatment for metastatic RCC. Individualized immunotherapy based on immunoreactive cytokines and/or antiangiogenetic agents (e.g. bevacizumab, sunitinib, and sorafenib) have showed encouraging results. Therefore, surgical resection should not be considered as the only therapeutic option: An interdisciplinary approach including visceral surgeons, urologists and oncologists should be performed for the treatment against pancreatic metastases to obtain sufficient synergistic antitumor effects. However, the best way to combine surgery with oncological treatment has to be evaluated addressed by future studies.

Pancreatic metastases can occur after a long disease-free interval, with a median time between resection of the primary tumor and detection of pancreatic metastases of 72 months (range, 0–228 months). This biological tumor behavior reflects the importance of a prudent long-term follow-up in these patients, even if specific symptoms are missing. This point is supported by our findings, which revealed that only 44.4 % of the patients had specific symptoms. We recommend that regular follow-up including radiological imaging should be performed even after a long disease-free interval.

Very few data are available regarding the potential impact of MVR for pancreatic metastases on morbidity and mortality because of the low proportion of patients with MVR in most of the published studies [20]. However, in our study, the majority of patients (66.6 %) were treated by a multivisceral approach. In a retrospective analysis, Strobel et al. compared patients who received either standard resection or MVR for pancreatic metastases of different tumor types [22]. The authors reported no significant difference for morbidity and mortality between groups, although morbidity in the MVR group tended to be higher. Also, the majority of complications were surgical, with one patient dying in each group. These results are comparable to our results where the majority of postoperative complications were also surgical. We also saw that major surgical complications occurred more frequently in the MVR group, although this result was not statistically significant. Because of its potentially beneficial impact on long-term survival, we do not consider that MVR is an absolute contraindication but that the increased operative risk should be considered in the decision making process. The overall morbidity in our study was not increased compared with the reported morbidity rate for resection of primary pancreatic malignancies of up to 58.5 %, even though major surgical complications occurred more frequently in our study [15]. This might be attributable to the high proportion of patients who underwent MVR.

During our study follow-up, only one patient suffered from tumor recurrence in the pancreas and this was successfully treated by resection of the pancreatic remnant without further tumor recurrence. This might be interpreted as a sign of good local tumor control and is supported by other studies [22].

In total, nine of our patients (50 %) developed extrapancreatic recurrence, of which five (33.3 %) had undergone previous MVR. Four patients received primarily combined liver and pancreas resection for metastases of sarcoma or gallbladder cancer (*n* = 1 for each cancer) and for metastases of malignant melanoma in two cases. In the latter patients, hepatic recurrence occurred and further surgical intervention was performed in one patient, who is still alive. These results are consistent with other reports showing a survival rate of 50 % after liver resection for malignant melanoma [23]. Therefore, the type of the primary tumor should also be considered when deciding on surgery and the patient should be informed of the risk of recurrence.

## Conclusions

A surgical approach with curative intent is justified in select patients suffering from metastases to the pancreas and offers good long-term survival. Our results showed that resection of pancreatic metastases of different tumor types was associated with favorable morbidity and mortality when compared with resection of the primary pancreatic malignancies. Our findings also demonstrated that MVR was feasible, with acceptable long term outcomes, even though morbidity tended to be higher than with standard resection.

## Abbreviations

DP: distal pancreatectomy
MVR: multivisceral resection
PPPD: pylorus-preserving pancreaticoduodenectomy
RCC: renal cell carcinoma
